# Basic and clinical immunology- 3019. Gamma interferon release assay for diagnosis of latent tuberculosis – comparison with TB skin test

**DOI:** 10.1186/1939-4551-6-S1-P195

**Published:** 2013-04-23

**Authors:** Prem Kumar, Robert O'brien, John Patterson, Chitra Desai

**Affiliations:** 1 Louisiana State University Health Sciences Center, Department of Medicine. Section of Allergy and Clinical Immunology, New Orleans LA, USA; 2 Louisiana Health Sciences Center, USA; 3 School of Medicine, Texas Tech University Paul L Foster, El Paso, USA

## Background

The presence of latent tuberculosis is routinely assessed by tuberculin skin test (TST), which detects a cell mediated immune response to the injected tuberculin purified protein derivative (PPD). Although TST has been in use for over a century, its limitations include poor specificity (i.e. numerous false positive results), the need to examine the site 48-72 hours after injection and the subjective interpretation of results (i.e. estimation of the diameter of induration). Recently, an in vitro assay has been investigated as an alternative to overcome limitations posed by TST. This assay, also known as gamma interferon release assay (GIRA) detects the release of gamma interferon from sensitized lymphocytes upon exposure to mycobacterium TB antigen coated tubes.

## Methods

The study included 17 healthcare workers ages 21 to 45 years who had positive TST as screening procedure for latent TB. In addition, 56 adult rheumatoid arthritis (RA) patients on anti TNF therapy were also included. TST were not performed on the immunosuppressed RA patients. GIRA was performed using ELISA technique involving whole blood utilizing mycobacterium TB antigen coated tubes along with positive and negative controls.

## Results

Twelve of the 17 healthcare workers were positive on TST and 5 had indeterminate TST results. Of the 12 subjects with positive TST results, 8 tested positive with GIRA testing (66.7%). However, 4 of the 12 with positive TST results were negative with GIRA testing (33.3%). Two of the 5 from TST indeterminate group tested positive with GIRA testing, while 3 were negative. All of the 56 immunosuppressed subjects tested negative with GIRA testing.

## Conclusions

This limited study reveals that TB skin test results can be falsely positive in as high as 33.3% of the people tested positive with TST. Additionally, GIRA testing is valuable to decipher those with TST indeterminate results. Therefore, GIRA is a superior method for diagnosis of latent TB and will reduce the unnecessary cost of treatment associated with false positive results with TST.

